# Micro-Foundations of Diverging Economic Policies: Keynesian, Behavioural, Neoclassic

**DOI:** 10.1007/s10272-020-0879-z

**Published:** 2020-04-01

**Authors:** Ronald Schettkat

**Affiliations:** Schumpeter School of Economics — VWL, Bergische Universität Wuppertal, Gaußstraße 20, 42119 Wuppertal, Germany

## Abstract

Germany’s austerity-oriented economic policy is the wrong approach. Markets need demand stimulation to achieve full use of resources, argues Krugman, a Keynesian economist. Neoclassical economists have been warning that expansionary macroeconomic policies are not only useless but can even be harmful (e.g. Phelps). These stark differences in the evaluation of economic policy proposals are deeply rooted in their underlying microeconomic reasoning, in the theories of the motivation and behaviour of economic agents as investors, workers, consumers and speculators as well as their interactions. The claim of missing micro-foundations in Keynes’s theory is false. In contrast, recent findings of behavioural economics have strongly confirmed Keynes’s micro-foundations that lead to his macroeconomic conclusions.

In Germany, austerity-oriented policy is regarded as a virtue and a condition for stable long-term growth. Even in the midst of the coronavirus crisis, Peter Altmaier, the Minister for Economic Affairs, promised to return to the 'black zero' soon. German economic policymakers believe that balanced budgets, the 'black zero' and huge net export surpluses are sacrifices made today that will pay off tomorrow (Brunnermeier et al., 2016). This is the wrong approach, argues Krugman (2019): slow growth is due to Germany's over-saving and austerity policies, creating not only problems for the German economy but also for other countries. Germany must increase its spending on consumption, education, public infrastructure, etc.[1] This will not help to overcome slow growth, counters Phelps (2019): structural reforms, increases in productivity growth and entrepreneurship are needed to revive growth in Germany and other industrialised economies. Microeconomics rather than macroeconomics is the solution.

## Diverging microeconomic foundations

Why do the propositions of the two Nobel-prize winning economists, Krugman and Phelps, deviate so much? Is it just the difference between macro- and microeconomics? Lucas and Sargent (1979) famously alleged that Keynes's theory was *intellectually flawed* because it misses micro-foundations. This allegation is *intellectually flawed*. Instead, Keynes's microeconomic foundations are the key to understanding his macroeconomics (Hahn, 1997). Keynes induced his micro-foundations from observed actual behaviour of entrepreneurs, speculators, workers and consumers in a monetary economy in which precautionary savings, speculations and investments are pursued in a dynamic environment under uncertainty. Behavioural economics is also based on this inductive methodological approach aimed at describing actual human behaviour. This approach contrasts sharply with the fictitious world deduced from neoclassical axioms.

The diverging micro-foundations and the concept of the economic environment are leading to fundamentally different equilibrium concepts – merely the balance between demand and supply in Keynes's theory, and the optimal equilibrium in neoclassical economics when individuals maximised utility and profits. In the equilibrium of the latter, all resources are fully used (full-employment equilibrium); no one can improve in the existing institutional framework through voluntary trade. There is no need for macroeconomic stimulation since the economy is already in the optimum (full-employment equilibrium); on the contrary, stimulation will destroy this optimal full-employment equilibrium, resulting in inflation only (Keynes 1936, 294). If the economy is assumed to be in full-employment equilibrium, improvements in growth and employment require shifts in the optimal equilibrium itself through, e.g. institutional reforms and the enhancement of productivity growth – just as Phelps suggested. However, the optimal equilibrium is an unverified assumption (Blinder and Solow, 1973).

1 A joint initiative of employers and unions argued in favor of higher investments in Germany, see Bardt et al. (2019)

**Ronald Schettkat**, Schumpeter School, Bergische University Wuppertal, Germany.

Keynes's theory does not exclude the optimal equilibrium, but the economy may balance very well below the optimum,[2] without any automatic forces to the full-employment equilibrium. To stimulate economic activity and overcome the stalemate, Keynes suggested expansionary macroeconomic policies (fiscal and/or monetary) to achieve full-employment equilibrium. The nature of the economic environment in which individuals operate as well as their behaviour distinguishes Keynes's theory from the axiomatic neoclassical model and results in the fundamentally different macroeconomic policy approaches.

This paper discusses the major commonalities between behavioural economics and Keynes's microeconomics as well as their distinction from neoclassical assumptions. The following sections discuss the analytical basis for the diverging views on the motivation of agents deduced from neoclassical axioms versus induced microeconomic theory. The research in behavioural economics impressively confirms Keynes's observations but contradicts neoclassical axioms.

## Motivation in economics

"All theory depends on assumptions which are not quite true. That is what makes it theory" (Solow, 1956, 65). Theoretical models stripped of irrelevant aspects should simplify the real world and help to better understand economic behaviour. Theories should work like glasses, but oversimplification may lead them to function instead like blinders. Does economic theory need to relate to the real world, or can theory be totally abstract from actual human behaviour? This is the fundamental methodological issue that distinguishes behavioural economics and Keynes's theory from the neoclassical model with far-reaching consequences. Both behavioural economics and Keynes regard economics as a real science such as physics or biology, where only assumptions that do not conflict with reality describe *the world we live in* (Kornai, 1971). Neoclassical economics is based on an axiomatic approach as applied in mathematical, logical sciences, in which *truth* is established through formal deductions from the axioms, which are not necessarily related to reality (Kornai, 1971). It assumes selfish, independent and socially isolated individuals who maximise their utility derived from real values (goods) along well-ordered and stable preferences using all available information (*rational choice*), subject to budget constraints. The interaction among individuals is limited to market exchange, i.e. choices made by other individuals leave their preferences unaffected; there are no trends, no fashions, no ostentation, no positional goods. Do individual motivation and decision-making follow the neoclassical axioms?

Doubts that actual decision-making processes are performed as required to maximise utility were expressed with an astounding *as-if* reply in the past (Friedman, 1953) and in the present (Pesendorfer, 2006). Axioms, Friedman (1953) argued, are instrumental,[3] and therefore theories cannot be judged by the validity of their axioms. On the contrary, he claimed, "Truly important and significant hypotheses will be found to have 'assumptions' that are wildly inaccurate descriptive representations of reality" (Friedman 1953, 8).[4] The only way to prove a theory is to determine whether its predictions are in accordance with the observed outcomes, but Friedman simply declared observed outcomes as optimal (utility- or profit-maxima), which is not the same as providing evidence. "When verification is demanded, they [neoclassical economists] tend to look for evidence that the theory makes correct predictions and resist advice that they should look instead directly at the decision mechanisms and processes" (Simon 1986, 38). Analysing the motivation and decisions of economic agents is precisely the research program of behavioural economics, aiming to develop a deeper and descriptive understanding of individual economic behaviour.

Everyone – including almost all economists – will admit to behaving irrationally[5] from time to time. However, most people regard their economic decisions as purposeful behaviour and have evaluated some pros and cons in order to reach the decision (at least if a more significant amount is spent). But *rational choice* means choosing the utility or profit-maximising alternative, which requires the intention to maximise as well as the ability to evaluate alternatives. *Rational expectations* require knowledge of all possible future events and their probabilities to calculate expected outcomes, which is a stochastic version of perfect foresight (Arrow, 1987). At best, rational expectations may apply to a steady-state economy where only external shocks can disturb the assumed smooth market process. The economy is treated like roulette, where the number of the next draw is unknown but where the probability distribution is known, i.e. while playing roulette is risky, one of the 37 possibilities must occur. However, is the economy actually moving along a smooth equilibrium path only disturbed by external shocks? Are expectations formed as mechanically as as-

2 Keynes's theory includes the optimal equilibrium as a special case. Therefore, he labelled his 1936 book, *The General Theory*.

3 Popper: Instrumentalism forces scientists to abandon the search for truth (cited according to Caldwell, 1980, 370).

4 The worse the assumptions, the better the theory? (F-twist, Samuelson, 1963).

5 Because *homo economicus* has dominated economics, many economists refer to other concepts of decision-making as irrational (e.g. Akerlof and Shiller, 2011). Giegerenzer and Selten (2001) distinguish rational theories based on known probabilities from non-rational theories, which are not about irrational decision-making. "Indeed, non-rational theories are concerned with psychological plausibility, that is, the capacities and limitations of actual humans, whereas rational theories have little concern for descriptive validity and tend to assume omniscience" (Giegerenser, 2001, 3).

sumed under rational expectations? Or are there interdependencies among the actions of economic agents? Lucas (1986) was prepared to narrow economics to the optimal equilibrium, to steady states, where expansionary macroeconomic impulses can only push the economy out of the optimum. Such policies can only be inflationary and ineffective because economic agents will anticipate (or learn to anticipate)[6] the outcomes within the Lucas model.

### Decisions under uncertainty: Animal spirits

*The economy we live* in differs sharply from roulette: the probabilities of future events are unknown and the future is not risky but uncertain as its growth path, its production potential, develops endogenously depending on expectations which cannot be *rational expectations*. How can consumers, workers and entrepreneurs decide under uncertainty? In the *real world*, as in Keynes's theory, expectations are radically different from *rational expectations* because the economic future is not a stochastic of perfect foresight but rather uncertain, i.e. even intended rational choices cannot be made.

Today, neoclassical economics simply builds models for variations of risk and ignores uncertainty as it did when Keynes was writing (Keynes, 1937, 212-213). Academic economists can ignore uncertainty and construct theories for an assumed certain or risky world (Arthur, 1994). But entrepreneurs make decisions in *the world we live in*, where uncertainty cannot be ignored, interdependencies are relevant as in financial markets, and where expectations are about the expectations of others (Keynesian beauty contest; Arrow, 1987)

The entrepreneur's production decisions depend on the expected demand, which in turn affects employment, labour income and demand; i.e. economic developments are endogenous – tomorrow's events depend on today's actions (Robinson, 1980; Howitt, 1986). Also, preferences may not be formed in isolation and are not fixed but may change under the influence of the decision of others. Economic agents, including businesspeople, must apply other guesswork, conventions, intuitions, emotions and *animal spirits* in order to decide. Clearly, the pros and cons will be evaluated before deciding on purchases and investments involving significant amounts. Possibly some probabilities may be assigned to certain outcomes, but uncertainty will remain and cannot be eliminated. Individuals can ascribe a certain probability to future events, but in an open, dynamic environment, these are bets (Dow, 2012). Keynes emphasises conventional behaviour (routines, heuristics) as an essential decision rule under uncertainty. Conventional behaviour or a conventional method of calculation assumes **"**that the existing state of affairs will continue indefinitely, except in so far as we have a specific reason to expect a change" (Keynes, 1936, 152). Economic agents rely on a limited number of heuristic principles (such as representativeness, availability, anchoring), intended to reduce complexity (Kahnemann, 2002, 465).

Under uncertainty, expectations are necessarily influenced by emotions, intuition, moods (optimism, pessimism) and *animal spirits*. Expectations and the following decisions also affect the present aggregate demand in the economy. Therefore, **"**a mere change in expectation is capable of producing an oscillation of the same kind of shape as a cyclical movement in the course of working itself out" (Keynes, 1936, 49). Furthermore, the instability of the economy becomes endogenous, as opposed to an assumed stable market system, in which only external shocks can disturb the optimal equilibrium. One feels more confident in estimating next year's sales than those ten or 20 years in the future for which investments are necessary. Although sales expectations for the next period are uncertain, the entrepreneur's confidence in these expectations is higher than in the long term. For decisions affecting the long term, confidence in the expected trends will be weaker, and the reasoning will be more elaborate, especially if investments are necessary, although uncertainty will remain. It cannot be eliminated.

Keynes's term *animal spirits* refers to the necessity to take action under uncertainty because *cold calculations* cannot bring about decisions.

> Most, probably, of our decisions to do something positive, the full consequences of which will be drawn out over many days to come, can only be taken as a result of animal spirits – of the spontaneous urge to action rather than inaction, and not as the outcome of a weighted average of quantitative benefits multiplied by quantitative probabilities (Keynes, 1936, 161).
>
> If human nature felt no temptation to take a chance, no satisfaction (profit apart) in constructing a factory, a railway, a mine, or a farm, there might not be much investment merely as a result of cold calculation (Keynes, 1936, 150).

Keynes's *animal spirits* are the basis for economic decisions under uncertainty but regarded with scepticism even among some Keynesian economists. "[I]f the animal spirits are dimmed and the spontaneous optimism falters, leaving us to depend on nothing but a mathematical expectation, enterprise will fade and die, though fears of loss may have a basis no more reasonable than hopes of profit had before" (Keynes, 1936, 162). Rationality is deeply ingrained in economists' thinking, but the work of neurolo-

6 See Schettkat and Jovicic (2017) for a discussion.

gist Damásio (1994) reveals that without emotions, without *animal spirits*, people are unable to decide. Damásio's discovery is based on the behaviour of a patient with brain damage in the prefrontal cortex where emotions are located, who functioned very well but could not make decisions. He would continuously collect information and evaluate alternatives, but would not use the information to make decisions.

The findings of behavioural economics reveal that humans often make systematic mistakes, even in predicting their own future utility. Sometimes humans make the wrong choices because they do not fully understand the situation or because they are not smart enough.[7] However, it is not simply an error or a misunderstanding of situations, which results in decisions deviating from rational choice. Even when misunderstandings are clarified, they stick to non-rational choices. It is not merely the *money illusion* that arises when nominal values are recognised in decisions; they can even overrule real values concerning utility.

## Non-rational economic decisions

Are we smart enough to choose rationally? Are our preferences as consistent as assumed in the neoclassical model? In an early experiment, Allais (1953) observed that the decision criterion under risk changes from expected payments to higher probability, resulting in contradictory choices between two lotteries (known as the Allais paradox). Participants in Allais's experiment had to choose between lotteries and voted (rationally) for the one with the highest expected value. When Allais changed the probabilities to one lottery with a guaranteed but lower gain compared to the other with a much higher expected value but also a minor chance of a zero gain, participants switched to the former. Obviously, certainty is preferred over higher gains with a slight probability of no gain.[8] Participants did not adhere to the axiom of rationality, and therefore the person's choices cannot be attributed to maximising the utility functions. Was it just a lack of understanding that caused the inconsistency of the participants' choices? Allais repeated his experiment at a conference with distinguished economics professors (among them Friedman, Samuelson, Savage and Arrow, according to Kahneman, 2011, 312-314). Even these distinguished fellows switched preferences as laypersons did, not noticing that their expressed preferences violated rational choice.[9]

A necessary condition for utility-maximising choices is accurate and unbiased forecasts of the hedonic outcomes of potential choices (Kahneman and Thaler, 2006). Many findings of behavioural economics suggest that the stable preferences assumption underlying the neoclassical model does not hold. To maximise utility requires correct knowledge of the utility that a specific product will provide. However, expected utility today and the utility experienced in the future might deviate substantially. People's (expected) utility seems to be strongly affected by the situation, their moods, the environment and time. **"**However, people do not always know what they will like, and they are likely to err most severely when the temporal gap is long and when the agent's state and circumstances vary between *t1* and *t0*" (Kahneman and Thaler, 2006, 223). To choose rationally economic agents must not only rank all possible choices, but they must also discount the future utility into present values, i.e. the measurement of utility needs to be cardinal. Furthermore, the future (expected) utility must be independent of current choices, but utility ascribed to a product may differ once a person owns the product (i.e. endowment effect, habituation, change of preferences, path dependence). Humans seem to value gains less than losses (prospect theory; Kahneman and Tversky, 1979). Participants in financial markets, presumably closest to the perfect market model (Schettkat, 2010), illustrate this behaviour: investors tend to keep shares even though the prices of which are falling.

A summary of 42 empirical studies on discount rates (Wilkinson, 2008, 314-317) found that discount rates do not rise with time as diminishing marginal utility would suggest but conversely decline with time. Humans seem not to apply exponential discounting (i.e. discounting with a constant discount rate), but rather apply hyperbolic discounting (Ainslie, 1991). A common finding in behavioural economics is that higher amounts are discounted at lower rates than smaller amounts (Kahneman and Tversky, 2000). Magnitudes influence the same person's discount rates but in the reverse order of the *rational choice* assumption: the higher the amounts, the lower the discount rate (Thaler, 1981).

## Socially embedded individuals

### Consumption

What determines consumption? Are the individual preferences influenced by the consumption and behaviour of others (i.e. positional goods, trends and fashion) or are prefer-

7 Thaler (2015) offers several examples in which humans simply make mistakes because the situation is too complex to be understood immediately.

8 Kahneman (2011) explains the switch with the certainty effect, under which the utility of a certain gain is substantially higher than the probability effect of a tiny deviation from certainty lowers the utility, which may make decisions sensitive to the presentation.

9 Kahneman (2011) mentions that Allais paradox was forgotten and became an anomaly with rationality remaining the standard assumption in economics.

ences formed in isolation and stable over time? The desires and needs of *homo oeconomicus* are not affected by trends, fashion and ostentation because one determines their preferences in isolation; only real income (the number of goods) counts and social interaction is excluded from the model. Neoclassical economics restricts the interaction among individuals to market exchange. For example, consumption of other individuals may affect relative prices (and via this mechanism, the choices), but the utility functions will remain unaffected.[10] This assumption is obviously not descriptive of the behaviour of social beings, but it is still alive and well in economics. "To many economists, the notion of consumers being strongly influenced by demonstration effects must have seemed probably inconsistent with the reasoned pursuit of self-interests, if not completely irrational" (Frank, 1985, 146).

Nevertheless, several economists took relative positions and social interactions into account: an early example can be found in the work of Veblen (1899).[11] For "any particular consumer will be more influenced by the consumption of people with whom he has social contacts" (Duesenberry, 1949, 48). Thus, signaling social status and demonstrating one's lifestyle through consumption is vital for socially embedded individuals (Leibenstein, 1975; Hirsch, 1976). Ostentation and extravagance are explicitly listed by Keynes (1936, 108) among the subjective factors influencing individual consumption. They are irrelevant for the socially isolated *homo oeconomicus*, who gains utility exclusively from real values (goods) and cannot place importance on the relative position of the income and consumption of others. Interdependent preference formation would destroy the basis for maximisation in the neoclassical model.

*Homo oeconomicus* is not influenced by her own history. She knows her preferences in advance, i.e. past consumption/income can serve as a reference point, leading to path dependence (habituation). Habitual standards of living involve judging situations by reference points, which depend on experience and the observed patterns of the reference groups (Baxter, 1988). These patterns themselves are affected by cultural influences, thus making preferences endogenous (Drakopoulos, 2011). Of course, within society, savings and consumption will be affected by the distribution of income.

## Real and nominal wages

Keynes observed that workers resist nominal wage reductions but accept similar real wage reductions through inflation. *Money illusion*, a lack of understanding, was and still is the common interpretation among economists who are used to thinking in models in which socially isolated individuals are solely stimulated by real wages (goods) and money is a veil covering the real values. This interpretation of Keynesian nominal wage resistance is totally ignorant of Keynes's reasoning that for social individuals, the position in society matters.

> [A]ny individual or group of individuals who consent to a reduction of money-wages *relative* to others will suffer a relative reduction in real wages, which is a sufficient justification for them to resist it. On the other hand, it would be impracticable to resist every reduction of real wages, due to a change in the purchasing power of money which affects all workers (Keynes, 1936, 14).[12]

Do workers care about relative wages? Probably the most robust evidence for the importance of relative pay comes from experiments using magnetic resonance imaging (MRT medical scanners): controlled for income levels, relative income is important and activates certain brain areas (Fliessbach et al., 2007). Labour contracts are stipulated in nominal terms, and these serve as a reference point. Reference levels (e.g. minimum wages) even serve as a reference point after they have been removed (Falk et al., 2006).

Strong evidence that money illusion is not the most relevant interpretation for nominal wage orientation is impressively revealed in a study by Shafir et al. (1997). Participants were presented with the cases of two individuals, A and B, who graduated simultaneously from the same college, got the same starting salary, but faced different pay increases and inflation. Participants were then asked to indicate which of the two is better off economically (in terms of real income), is happier and would be more likely to quit upon receiving an alternative job offer. Figure 1 shows the outcome of this experiment. Assuming a rational individual, person A should be happier because she got a higher real income, and consequently, she should be less likely to quit. However, the majority of respondents in the study understood very well that person A is better off economically; i.e. participants do not suffer from money illusion, but they nevertheless believe that person B is happier and less likely to quit.

Nominal values, money wages and their changes are obviously regarded as important by most people, even if the real wage is lower than in the alternative. This result sheds

10 Elsner (2012) enhances the traditional microeconomic analysis to complex interactions.

11 Frank (1999) argued that the consumption of the neighbours, the Joneses, as a reference was substituted by the consumption of the very wealthy through mass media, which arguably leads to overconsumption.

12 Workers usually cannot afford to withdraw their labour in response to nominal wage cuts, but they can reduce their effort (Bewley, 1999).

light on a conundrum in labour economics, where it was found that upward-sloping wage profiles are not necessarily related to productivity improvements (Medoff and Abraham, 1981). When workers are offered the choice between a rising wage profile with a lower starting income and a declining wage profile with a higher starting income, the majority chose the former, even if the advantage of declining wage profiles is explained to them (Loewenstein and Sichermann, 1991).

## The impact of endogenous preferences

The valuation of situations related to reference points is arguably the most crucial finding in behavioural economics, especially in the context of methodological concerns. The endowment effect (Kahneman et al., 1991) refers to changes in the valuation of products, depending on the possession of a product. When buying a product, its utility should be equal to the utility derived from the money that was paid for it (respectively to the alternative products the money could have bought). However, it turns out that, once a product is possessed, it is only sold at a significantly higher price (although the owner would not have paid the potential selling price). Such behaviour has been observed for football and concert tickets (Krueger, 2001), bottles of wine and even coffee mugs. The utility derived from the possession of the product rose after the consumer possessed it. In other words, preferences are endogenous and are evaluated asymmetrically, as summarised by prospect theory (Kahneman and Tversky, 1979). Kahneman and Tversky found that the utility gain of adding an additional unit of a certain product is less than the utility loss of subtracting a unit of the very same product would be. The orientation on reference points implies changes in the utility function, i.e. the neoclassical assumption of stable preferences is violated.

Komlos (2014) translated the impact of prospect theory into conventional indifference curves and shows that, after initial maximisation (in which the budget constraint is tangential to an indifference curve), kinks will arise at the reference point because of the differing valuation of the gains and losses. The marginal rate of substitution between two goods X and Y ($m = -dY_i/dX_i$) changes, because the loss in Y weighs greater than the gain in X. In other words, the slope of the indifference curve changed, even if the maximisation procedure was initially performed. In a new situation, however, asymmetric evaluation will repeat. Therefore, neoclassical theorist Pesendorfer (2006) concludes that a rational economic agent, knowing that a decision changes her preferences, will refrain from maximisation. In other words, a rational economic agent does not follow rational decision-making because the basis for it does not exist – *super rationality*.

Figure 1

**Orientation on nominal versus real salaries: Money illusion?**

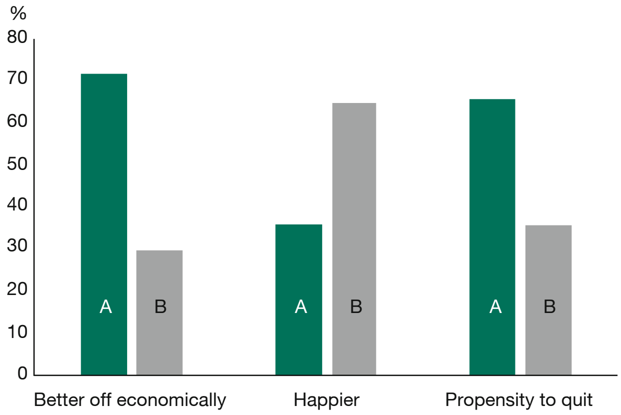

Note: Both persons started after graduation, simultaneously, with the same income. Person A receives a pay rise of 2% in times of no inflation, while person B receives a 5% pay rise in times of 4% inflation.

Source: Compilation from Shafir, E., P. Diamond and A. Tversky (1997), Money illusion, *The Quarterly Journal of Economics*, 112(2), 341-374, cited in Wilkinson, N. (2008), An introduction to behavioral economics, Palgrave Macmillan.

## Conclusions

The allegation that Keynes's theory is flawed because it misses micro-foundations is itself flawed. On the contrary, Keynesian micro-foundations are induced from observed economic behaviour in *the world we live in* and are strongly confirmed by the findings of behavioural economics. Keynes and behavioural economics share an inductive methodological approach that refers to *the economy we live in*, and that allows for socially embedded individuals. The experiments and tests in behavioural economics extend far beyond Keynes's *casual* observations, providing rigorous evidence for regularities in economic human behaviour which contradict neoclassical axioms and should not be ignored. Furthermore, *the world we live in* is not a risky move along a steady state path, but it is uncertain with the consequence that expectations cannot be based on cold calculations, they cannot be calculated rational expectations; instead, they are endogenous and made under uncertainty. Today's expectations and decisions affect tomorrow's outcomes. Since behavioural economic findings contradict the neoclassical axioms, it may provide the seeds of a scientific revolution in the Kuhnian sense (Kuhn, 1970).[13] These seeds, within broader, more realistic economics, were already underlying Keynes's micro-foundations of his macroeconomics.

13 For a discussion of whether behavioural economics is an extension or an alternative to the neoclassical approach, see Schettkat (2018).
